# American Pharmacy Abroad

**Published:** 1883-11

**Authors:** 


### AMERICAN PHARMACY ABROAD.

The aggressiveness of American enterprise received a very striking illustration at the late International Pharmaceutical Exhibition held at Vienna, in the display of products from the laboratory of our energetic countrymen, Messrs. Parke, Davis & Co., of Detroit, Michigan. We notice in the reports as published in the domestic journals, and from the special correspondence of foreign journals, that this display while exciting much interest from its scientific features, attracted more than ordinary notice from its artistic beauty and finish among the non-professional visitors. This lay interest was, doubtless, largely due to the special attention given the display by the Emperor and the Archduke Karl Ludwig. These royal visitors manifested unusual interest in this exhibition of American taste, and took occasion to especially compliment the firm, through its representative, on its enterprise and skill. We congratulate Messrs. Parke, Davis & Co. on the distinguished recognition of the artistic excellence of their laboratory products. Their intrinsic worth needs no commendation from us ; this has long been conceded by the profession. The gold medal awarded them by the Vienna Exhibition is but an indorsement of the esteem in which this scientific commercial house is held in this country, where it is best known.

